# The Impact of Moderate Earthquakes on Antidepressant Prescriptions in Ulsan, South Korea: A Controlled Interrupted Time Series Analysis

**DOI:** 10.2188/jea.JE20220171

**Published:** 2023-12-05

**Authors:** Eun-San Kim, Jiyoon Yeo, Yongjoo Kim, In-Hyuk Ha

**Affiliations:** 1 Jaseng Spine and Joint Research Institute, Jaseng Medical Foundation, Seoul, Republic of Korea; 2 Department of Economics, Korea University, Seoul, Republic of Korea; 3 College of Korean Medicine, Sangji University, Wonju, Republic of Korea

**Keywords:** earthquake, mental health, antidepressant, interrupted time series analysis

## Abstract

**Background:**

In 2016, two consecutive moderate magnitude earthquakes occurred in Ulsan, South Korea. Therefore, we aimed to investigate the impact of earthquakes on the mental health of residents in Ulsan.

**Methods:**

We used data from the 2015–2017 Korean Health Insurance Review & Assessment Service National Patient Sample. We conducted an interrupted time series analysis using location-based controls. Changes in the number of antidepressants, benzodiazepines, and zolpidem prescriptions in Ulsan were compared to controls. Overall changes in weekly prescriptions 1 year after the first earthquake, compared to a non-earthquake scenario, were estimated.

**Results:**

In antidepressant prescriptions, the increase in trend after an earthquake was significantly higher than controls. However, the changes in benzodiazepines and zolpidem prescribing were not significant. Overall, the impact of the earthquake on weekly antidepressant prescriptions at 1 year was estimated as a 1.32 (95% CI, 1.18–1.56) rate ratio compared to the non-earthquake scenario. This corresponded to an increase of 1,989.7 (95% CI, 1,202.1–3,063.0) in the number of prescriptions. Among subgroups, the increase was highest among males aged 20–39 years.

**Conclusion:**

The moderate earthquake in Ulsan was associated with an increase in antidepressant prescriptions. The increase in the male group aged 20–39 was the highest. The impact may vary according to the context of the population.

## INTRODUCTION

In 2016, two consecutive earthquakes occurred in Ulsan, South Korea. On July 5, 2016, a 5.0-magnitude earthquake occurred 52 km east of Ulsan. In Ulsan, this earthquake had an intensity of IV on the Modified Mercalli Intensity scale (MMI). After 2 months, a 5.8-magnitude earthquake, with an MMI scale of V, occurred approximately 25 km northwest of Ulsan. To date, these remain the most powerful earthquakes in Korean history. A total of 23 residents from Ulsan and nearby regions were hospitalized, and four of them were Ulsan residents. No fatalities were reported. The earthquakes mainly caused property damage, resulting in 5,664 houses and about 10 million dollars’ worth of properties being affected. Some regions suffered from the aftermath of the earthquakes, such as plummeting tourism based on the fear of earthquakes. Moreover, misleading information was spread through social media, and there was fear that more severe earthquakes would strike shortly thereafter.^1^

The impact of earthquakes on mental health is widely known. Earthquake victims are vulnerable to several mental health problems, such as posttraumatic stress disorder (PTSD) and depression.^2^^–^^4^ Moreover, several risk factors for mental health problems (including gender and age) have been studied.^2^^,^^5^^,^^6^ Although previous studies have focused on major earthquakes (those with a magnitude ≥6)^2^^–^^6^ and the number of victims is incomparable even with moderate magnitude,^7^ they have suggested that loss of properties, house damage, and low economic status are associated with mental health problems.^2^^,^^5^ This suggests that even non-fatal moderate earthquakes can negatively affect mental health. Thus, we conducted research to investigate the impact of moderate magnitude earthquakes on residents’ mental health in Ulsan.

Due to the nature of natural disasters, it is difficult to precisely estimate their impact on mental health. Earthquakes have an impact on the residents in the regions extensively; thus, it is difficult to recruit representative samples for entire regions. Similarly, it is difficult to recruit control groups, which are essential for causal estimates. To deal with these challenges, we used nationally representative healthcare data with a controlled interrupted time series (CITS) model.

The interrupted time series (ITS) model has been used for studies wherein randomization is not feasible.^8^ In an interrupted time series model, the difference in observed and expected values is estimated. Expected values are estimated based on the pre-existing trend and represent the ‘counterfactual’ values that could have been observed if the events did not happen. Thus, the difference in observed and expected values after an event translates to the effects of the event. The differences can be modeled with various measures, such as level and trend changes. ITS analysis is used in wide ranges of studies, such as in public health interventions at the national level^9^^,^^10^ or for research on natural disasters.^11^^,^^12^ However, the ITS can be biased with time-varying confounders, which occur concurrently with interventions. By comparing the changes with relevant controls, CITS can estimates unbiased effects of event.^13^ In this way, we aimed to investigate the impact of earthquakes on the mental health of people in Ulsan.

## METHODS

### Data

We used data from the 2015–2017 Korean Health Insurance Review & Assessment Service - National Patient Sample (HIRA-NPS). The validity and reliability of this sample have been previously established.^14^ The HIRA-NPS was constructed via the stratified sampling of 3% of all patients using national health insurance-covered medical services over 1 year (approximately 1.4 million patients). We applied the weights in the analysis according to this sampling strategy.

The Korean National Health Insurance is a single-payer system, so the HIRA-NPS represents the whole population in Korea. The HIRA-NPS contains the basic demographic information of the patients, such as gender and age, as well as information on the medical services provided, including drug prescriptions. Furthermore, the HIRA-NPS provided information on the location of medical institutions where patients were prescribed medications. Using this information, we identified the study population.

### Definition of the event

**Figure 1.**
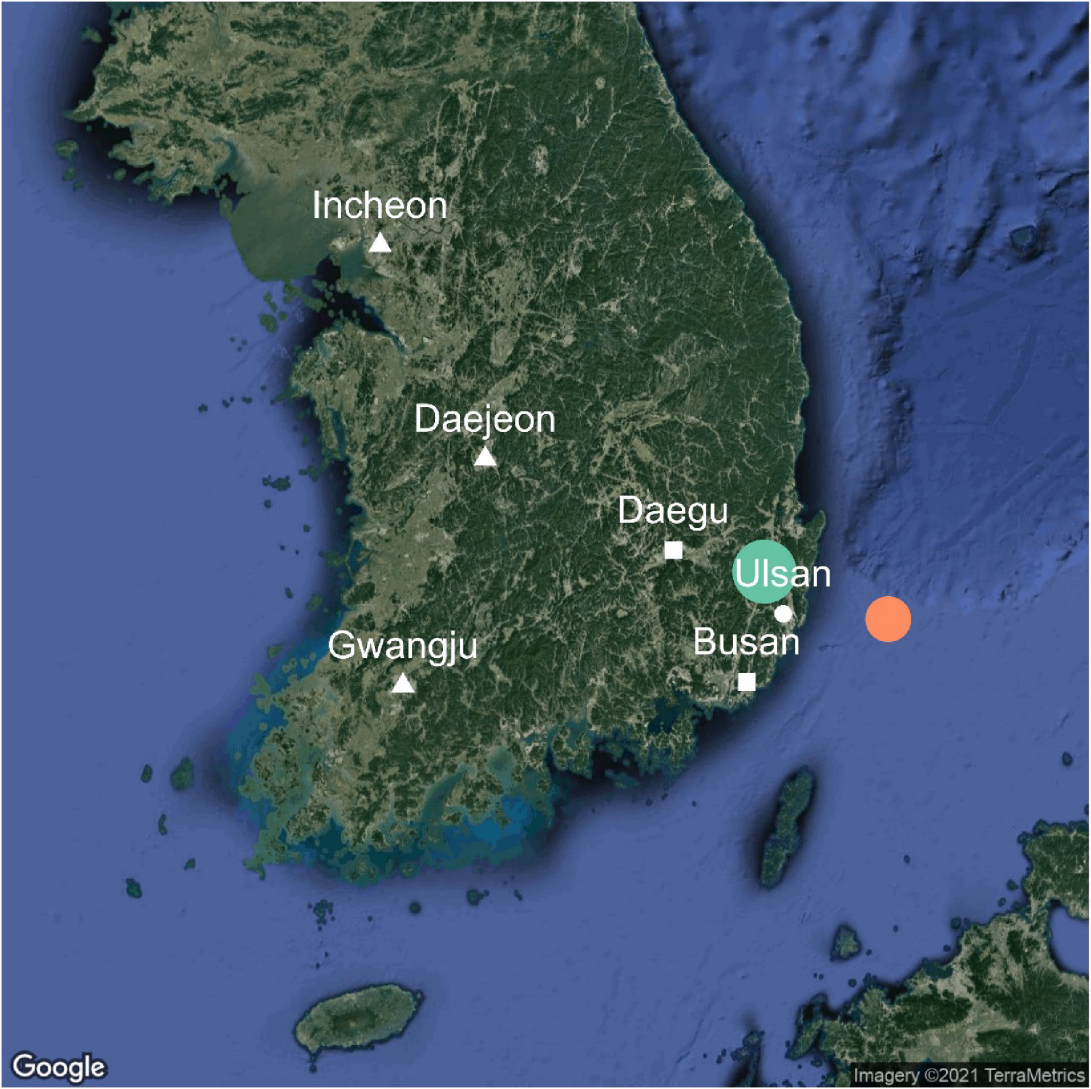
The distribution of earthquakes with a magnitude of 5.0 or higher across South Korea, from February 25, 2015 to November 14, 2017. The first earthquake (with a magnitude of 5.0 and an MMI intensity of IV in Ulsan) occurred on July 5, 2016. It occurred 52 km east of Ulsan (red circle). The second earthquake (with a magnitude of 5.8 and an MMI intensity of V in Ulsan) occurred on September 12, 2016. It occurred in about 25 km northwest from Ulsan (blue circle). The white circle represents the location of Ulsan. The other white shapes represent the cities that can be compared to the Ulsan considering the administrative divisions of South Korea. Incheon, Daejeon, and Gwangju (triangles) were scarcely affected by the earthquakes analyzed during the study period. Incheon was selected as a control group in the main analysis.

The regional distribution and magnitude of the analyzed earthquakes are represented in Figure 1. The first earthquake occurred on July 5, 2016; it had a magnitude of 5.0 and an intensity of IV on the MMI scale in Ulsan. The second earthquake occurred on September 12, 2016; it had a magnitude of 5.8 and an MMI scale of was V in Ulsan.

We assumed that Ulsan residents were affected, as the first earthquake struck the region on July 5, 2016. Both earthquakes occurred within a short period (2 months); the correlation of time from events was 0.99 (Pearson correlation coefficient: *r* = 0.99; *P* < 0.001). Therefore, it was almost impossible to analyze the effects of the earthquakes separately. Moreover, although the second earthquake’s MMI scale was higher, the fitness of the model was better when it was modeled based on that Ulsan residents were affected by the first earthquake. The consistency of our results was tested via sensitivity analysis by defining the event differently.

Ulsan residents were observed until November 14, 2017, a day before a third earthquake (5.4 magnitude/IV MMI intensity) struck near Ulsan. The pre-earthquake period was defined as long as the post-earthquake period (497 days). Hence, the starting date for the pre-earthquake period was set to February 24, 2015.

### Controls

We used location-based controls.^13^ According to the administrative divisions in Korea, Ulsan is classified as a metropolitan city (Gwangyeoksi). A metropolitan city constitutes the upper level of local autonomy in Korea. The population of a metropolitan city, which is highly developed city, is at least one million. Therefore, the controls were selected from other metropolitan cities.

There are five other metropolitan cities in Korea. Of these, Daejeon, Gwangju, and Incheon were outside the impact radius of the two earthquakes. The earthquakes did not have sufficient magnitude to affect these cities during the follow-up period either (Figure 1). Gwangju was selected as our control because pre-trend before earthquake was most similar among metropolitan cities (pre-trend differences were all non-significant). A comparison with the other metropolitan cities mentioned above was conducted as sensitivity analysis. The population characteristics of Ulsan and controls are presented in eFigure 1.

### Outcomes

The outcome was defined as changes in the prescription of antidepressants, benzodiazepines, and zolpidem. Changes in antidepressant prescriptions are widely used as a measure of the impact of disasters on mental health at the community level.^11^^,^^15^^,^^16^ Benzodiazepines and zolpidem cover most of the anxiolytics and hypnotic/sedative medications prescribed in Korea.^17^ The prescription of each medication was identified according to the Anatomical Therapeutic Chemical code (antidepressants: N06A; benzodiazepines: N05BA, N05CD; zolpidem: N05CF02; Details are described in eTable 1). The number of prescriptions was summarized weekly per each subpopulation of each metropolitan city.

### Analysis

As explained above, we conducted a controlled interrupted time series analysis to investigate the impact of the earthquakes on prescription rates (detailed explanation of the model is presented in eMaterial 1). In short, we examined the level and trends of changes with a ratio of rate ratios (RRR). Due to the different sizes and structures of the populations of Ulsan and the controls, we concluded that using standardized rates would be appropriate for comparison. Later, we conducted single ITS in Ulsan only. The changes were confirmed when they are significant in both CITS and ITS.^13^ After confirming the changes in prescriptions, we estimated the overall rates and absolute volume changes in the weekly prescriptions 1 year after the first earthquake, compared to a non-earthquake scenario.

Sensitivity analyses were performed. First, other metropolitan cities (ie, Daejeon and Gwangju) were used as controls. Second, to verify that prescriptions in control regions were not affected by the earthquake, we performed single ITS with control regions. Third, we assumed that Ulsan residents were affected since the second earthquake, on September 12, 2016. Fourth, since January 1, 2017, permission for antidepressant prescriptions was partially extended. Before 2017, non-psychiatrist physicians were prohibited from prescribing antidepressants for more than 2 months.^18^ The restriction was eased, so that non-psychiatrists could prescribe antidepressants for depressive symptoms for more than 2 months if the patients had cerebrovascular disease, epilepsy, dementia, or Parkinson’s disease. We conducted a sensitivity analysis that excluded antidepressant prescriptions for patients with cerebrovascular disease, epilepsy, dementia, or Parkinson’s disease. In the subgroup analysis, we investigated the heterogeneity of the impact according to Ulsan residents’ gender and age. All analyses were conducted using R studio software, version 1.4.1103 (R Foundation for Statistical Computing, Vienna, Austria).

### Ethical statements

The study protocol was approved by the institutional review board of the Jaseng Hospital of Korean Medicine (JASENG 2021-06-003) and followed relevant guidelines. The requirement to obtain participants’ informed consent was waived by the institutional review board.

## RESULTS

**Figure 2.**
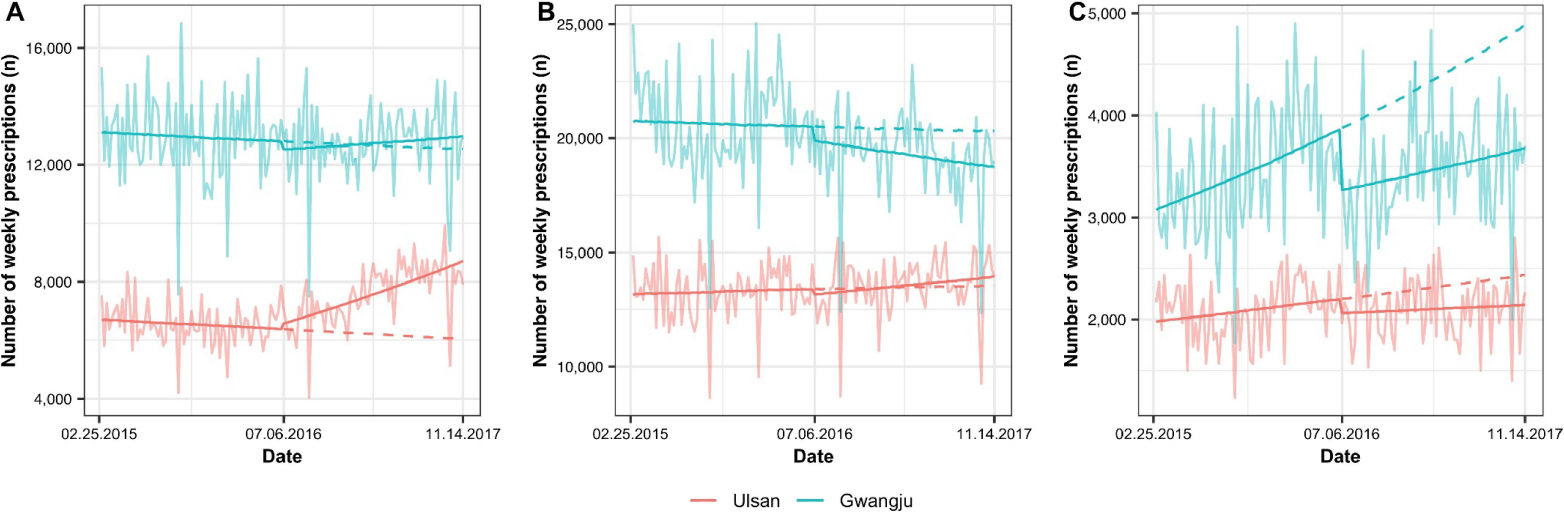
Observed and estimated volume of weekly prescribed psychotropic medications in Ulsan and in the control region during the study period. The first earthquake occurred on July 5, 2016. The solid line is modelled data and the dashed line is counterfactual data with assumption that the earthquakes did not occur. The modelled data are de-seasonalized. Translucent lines are observed data. The trend changes in control group were all non-significant. A: antidepressants; B: benzodiazepines; C: zolpidem.

The number of psychotropic medication prescriptions during study periods is presented in Table 1. In antidepressant prescriptions, the increase in trend after the earthquake was significant, compared to the control (RRR 1.003; 95% CI, 1.002–1.005; eTable 2). However, the change in benzodiazepines was not significant and only level increase in zolpidem was significant (RRR 1.111; 95% CI, 1.019–1.243). The observed and modeled prescription changes are presented in Figure 2. In our sensitivity analysis, the results in antidepressants prescriptions were consistent (see eTable 2 and eFigure 2, eFigure 3, eFigure 4, and eFigure 5). Moreover, there were no significant antidepressants prescriptions changes in control regions (eTable 3).

**Table 1.** Number of psychotropic medication prescriptions before and after the first earthquake in Ulsan residents, subpopulations of Ulsan, and controls

		Antidepressants		Benzodiazepine		Zolpidem	
		Pre-earthquake	Post-earthquake	Pre-earthquake	Post-earthquake	Pre-earthquake	Post-earthquake
**Ulsan**	Total	464,761	538,761	943,255	961,556	148,265	149,165
**Subpopulations**							
Sex	Female	284,497	325,097	579,360	575,394	90,466	93,699
	Male	180,264	213,664	363,896	386,162	57,799	55,466
Age, years	<20	14,867	18,900	19,900	19,066	567	300
	20–39	78,433	83,499	144,499	133,698	16,267	16,866
	40–59	172,698	226,431	389,229	415,963	67,066	61,099
	≥60	198,764	209,931	389,628	392,828	64,366	70,899
**Controls**							
Incheon	Total	1,105,020	1,211,320	2,145,274	2,132,676	553,927	563,827
Gwangju	Total	919,355	904,089	1,464,548	1,370,118	245,097	246,130
Daejeon	Total	1,030,320	1,063,054	1,789,511	1,694,280	292,930	272,730

The first earthquake in Ulsan occurred on July 5, 2016. The pre-earthquake period ranged from 2015-02-24 to 2016-07-05. The post-earthquake period ranged from 2016-07-06 to 2017-11-14.

**Figure 3.**
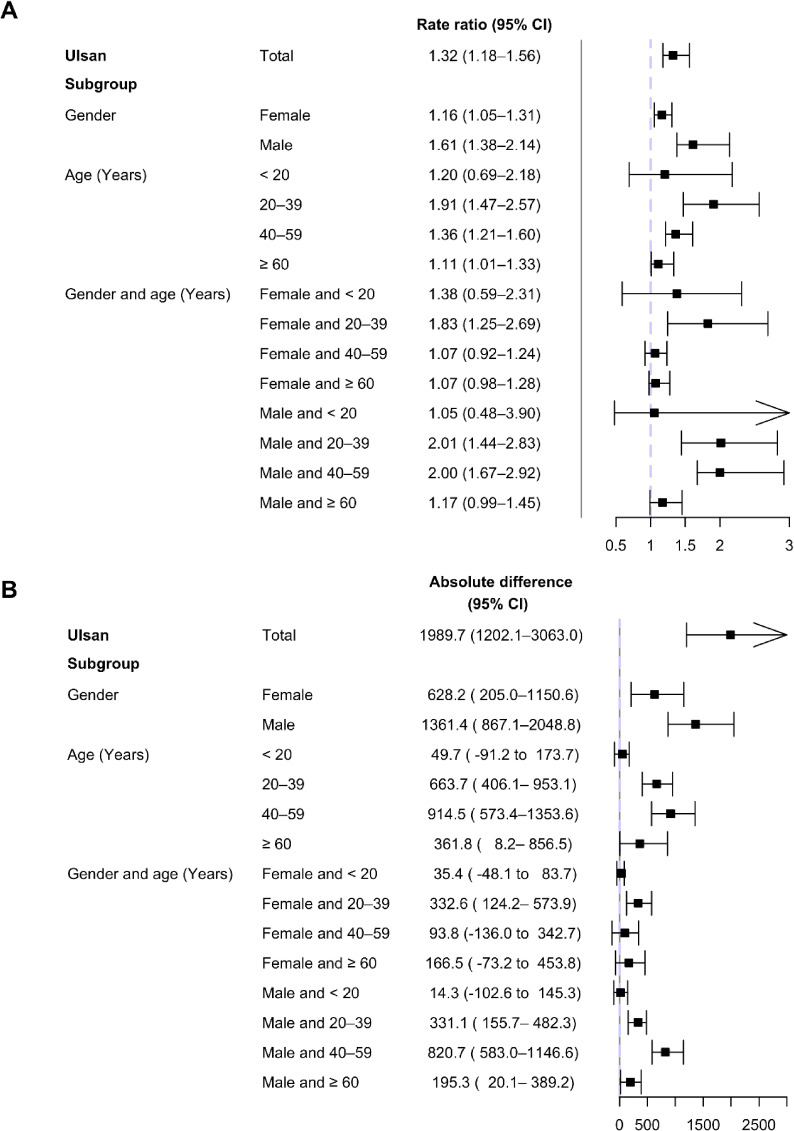
Changes in rate ratio and absolute volumes of weekly antidepressant prescriptions in Ulsan and its subpopulation 1 year after the first earthquake. Overall changes in weekly antidepressant prescriptions 1r after the first earthquake are presented as a rate ratio and absolute volumes. The amounts of absolute volumes are scaled to present whole population changes. The interactions with subpopulations are all significant.

In single ITS, trend change was significant (RR 1.005; 95% CI, 1.003–1.007) and results were generally consistent in subgroups (see eTable 4). The overall increase in weekly antidepressant prescriptions in the RR, compared with the non-earthquake scenario, was 1.32 (95% CI, 1.18–1.56) 1 year after the earthquake. This corresponded with a 1,989.7 (95% CI, 1,202.1–3,063.0) increase in the absolute volumes. Among subgroups, the overall change in the male population (RR 1.61; 95% CI, 1.38–2.14; absolute volumes: 1,361.4; 95% CI, 867.1–2,048.8) was higher than that in the female population (*P*-value of interaction <0.001). Considering both genders and all age groups, the RR was highest among men aged 20–39 years (RR 2.01; 95% CI, 1.44–2.83; absolute volumes: 331.1; 95% CI, 155.7–482.3), followed by men aged 40–59 years (*P*-value of interaction <0.001; Figure 3).

## DISCUSSION

We found that the earthquakes in Ulsan were associated with an increase in antidepressant prescriptions. After the first earthquake, an increased trend in weekly antidepressant prescriptions was observed in both CITS and ITS. Overall, the impact of the earthquakes on weekly antidepressant prescription rates 1 year later was estimated as RR 1.32. The impact of the earthquakes varied according to sex and age, being worst among men aged 20–39 years.

To the best of our knowledge, this study is the most robust evidence of the impact of moderate magnitude earthquakes on mental health. The magnitudes and numbers of victims analyzed in previous studies are not comparable to those of the Ulsan earthquake.^2^^–^^4^^,^^7^^,^^19^ In Korea, two surveys were conducted for residents who experienced similar-scale earthquakes in other regions^20^^,^^21^; however, due to their design, the representativeness of their sample was restricted and no controls were recruited. In this study, we used a nationally representative Korean sample. Further, we conducted a controlled interrupted time series analysis for causal estimates.

However, the increase in benzodiazepines was not significant and level change in zolpidem lacked robustness. In a previous study investigating the impact of disasters on mental health in Korea, the results were similar. Han, Kim, Lee, Lee, Ko and Paik^15^ studied the impact of the Sewol ferry disaster, in which 304 of 476 passengers were reported missing or deceased and measured the changes in psychotropic medication prescriptions in Ansan, Korea, where many of the victims lived. The antidepressant prescription rates increased after the disaster; however, rates of prescription for anxiolytics and hypnotic/sedatives did not. The authors claimed that this result might be related to benzodiazepine-related public policies in Korea. The inappropriate usage of benzodiazepine has been an issue in Korea. The most frequent inappropriate prescription among older adults in Korea was benzodiazepine.^22^ Also, there have been numerous studies investigating possible adverse events associated with benzodiazepine use in Korea.^23^^,^^24^ Due to this problem, the Korean government introduced several measures to reduce benzodiazepine prescriptions; for example, prescribing periods of benzodiazepines are restricted (<30 days per 1 prescription). There has been a slight decrease from 2009 to 2013.^25^ Also, since October 1, 2015, the drug utilization review (DUR) was implemented. The Ministry of Food and Drug Safety in South Korea introduced DUR to suppress the prescription of tricyclic antidepressants and long-acting benzodiazepine use in older adults aged 65 years or more. Under the DUR system, prescribers receive real-time caution messages on their computer if they prescribe inappropriate medications to outpatients aged 65 years or more. The message warns about possible adverse events and suggests that these medications should be administered in low doses.^26^ These policies might have prevented the increase in benzodiazepines. However, we have no clear explanation for these results yet.

We were unable to analyze the additional effects of the second earthquake on September 12, 2016, because both earthquakes occurred so closely in time. Although the results became clearer when our model was based on that the residents were affected since the second earthquake, this does not mean that we analyzed the impact of the second earthquake separately. Considering that the damage of the second earthquake was more severe than that of the first, it would seem likely that the second earthquake aggravated the impact of the first. However, we do not think this possibility is a limitation. Since both earthquakes occurred consecutively, they can be considered as one event. Moreover, given that previous disasters have also occurred in quick succession,^2^^,^^4^ this does not reduce our findings’ generalizability.

The results of the subgroup analysis contrasted with those of previous studies. The antidepressant prescription rates increased most among men aged 20–39 years. In previous studies, women have been reported to be more vulnerable to earthquakes’ effects on mental health.^2^^,^^5^^,^^6^ In contrast, some of our findings were consistent with previous studies, where younger and middle-aged people were more vulnerable than older individuals.^6^^,^^27^^,^^28^ This may be attributable to the economic burdens on the working-age population.^6^ Also, in the case of other disasters, economic crises, such as recessions, have been known to negatively affect the male population’s mental health.^29^^–^^31^ Accordingly, this may explain why the earthquakes had a greater impact on males. Ulsan is a manufacturing-oriented society. The proportion of males among the economically active population in Ulsan is the highest among the metropolitan areas in Korea.^32^ As mentioned, the earthquakes mainly affected the economy; houses and properties were damaged and tourism in nearby regions suffered due to the fear of earthquakes (eg, Gyeongju city).^1^ Consequently, the economy in Ulsan and the adjacent areas was also negatively affected. Thus, it can be assumed that the ensuing economic burden might be stressful to the working population, which is mainly composed of males. However, the data used in this study did not include the economic status of the patients, so this aspect should be explored in future studies.

Media influence might have aggravated the effects of the earthquakes on mental health. After the events, there were rumors and speculation that more severe earthquakes that would quickly follow. Images of the disaster and unverified theories were spread through social media,^1^ and this media exposure may have negatively affected mental health.^33^ For example, in the case of the Sichuan earthquake in 2008, frequent exposure to images of the disaster was associated with a higher likelihood of PTSD.^34^ Similar cases have been reported for terrorism^35^ and pandemics.^36^ Moreover, rumors on social media can exaggerate the threat and increase mental stress.^37^ Our results indicated that the prescription rate increased significantly in the 20–39 age group, who are frequently exposed to social media. Thus, media coverage of the earthquakes might have attributed to the results; however, further studies are needed.

As to the generalizability of our findings, the following characteristics of Ulsan should be considered. Ulsan is a highly developed industrial city. Further, its prescription rates for psychotropic medications were relatively lower than those of other metropolitan cities. These characteristics could affect attempts to generalize our findings. For example, if earthquakes of equivalent magnitude occur in different regions with a greater number of economically underdeveloped residents and patients with mental diseases, the impact might be more severe. On the other hand, considering the more prominent medical infrastructure in Ulsan compared to neighboring regions, it can be assumed that patients from nearby regions traveled to Ulsan for medical purposes. Visiting bigger cities for healthcare utilization happens frequently in Korea.^38^^–^^40^ Moreover, it takes only an hour by car to reach Ulsan from Gyeongju city, where the residents also suffered from earthquake damage. Thus, Ulsan represents damage to both Ulsan and the nearby regions.

The major limitation of this study is that we did not analyze the incidence of mental diseases, which is the primary indicator of mental health. Moreover, we did not analyze the diseases for which the psychotropic medications were prescribed. In Korea, there is a significant social stigma attached to mental disease, a tendency that varies according to sex and age.^41^^,^^42^ Moreover, psychotropic medications, such as antidepressants, are prescribed for a variety of conditions, not only for depression and PTSD but also for pain management.^43^^,^^44^ Thus, even if the patients exhibited mental health issues, it is likely that some of them were prescribed psychotropic medications under different diagnosis. Therefore, we concluded that investigating overall prescription rates would be appropriate in the present design. These limitations should be investigated in further studies.

We believe that our results are robust despite some major events regarding antidepressant prescriptions in South Korea. First, as explained above, the DUR was implemented on October 1, 2015. DUR aimed to suppress the prescription of tricyclic antidepressants in older adults aged 65 years or more.^26^ However, DUR was implemented in both Ulsan and controls and total antidepressant prescription was on the rise despite the DUR implementation. Thus, it seems that DUR had little effect on our results. Second, since January 1, 2017, restriction on antidepressant prescriptions was eased for patients with cerebrovascular disease, epilepsy, dementia, or Parkinson’s disease. Our sensitivity analysis indicates that the results are robust regardless of the policy change.

### Conclusion

In conclusion, the moderate magnitude earthquakes that occurred in Ulsan, South Korea in 2016 were associated with an increase in antidepressant prescriptions, especially among men aged 20–39. The impact of the earthquake may vary according to the context of the population.

## SUPPLEMENTARY MATERIAL

eTable 1. The list of psychotropic medications included in the study and corresponding anatomical therapeutic chemical code

eTable 2. Estimated level and trend changes in weekly psychotropic medications prescriptions in ratio of rate ratios compared to the controls

eTable 3. Estimated level and trend changes in weekly psychotropic medications prescriptions in controls

eTable 4. Estimated level and trend changes in weekly antidepressant prescriptions in rate ratio and absolute volumes in Ulsan compared to non-earthquake scenario

eFigure 1. Population characteristics of Ulsan and controls at the start (February 2015) and the end (November 2017) of the study period

eFigure 2. Observed and estimated volume of weekly prescribed psychotropic medications in Ulsan and control (Daejeon) during the study periods

eFigure 3. Observed and estimated volume of weekly prescribed psychotropic medications in Ulsan and control (Incheon) during the study periods

eFigure 4. Observed and estimated volume of weekly prescribed psychotropic medications in Ulsan and control during the study periods with a different way of defining the event

eFigure 5. Observed and estimated volume of weekly prescribed antidepressant in Ulsan and controls during the study periods excluding patients with cerebrovascular disease, epilepsy, dementia, or Parkinson’s disease

eMaterial 1. Details on statistical analysis
