# Supplementary material for: The Impact of Moderate Earthquakes on Antidepressant Prescriptions in Ulsan, South Korea: A Controlled Interrupted Time Series Analysis
Source: J Epidemiol. 2023 Dec 5;33(12):600–6. doi: 10.2188/jea.JE20220171 (PMC10635813; doi:10.2188/jea.JE20220171)
Supplement: Supplementary file 1 [file je-33-600-s001.zip › JE20220171/JE20220171_eMaterial_accepted_33-12-clean.pdf]

eMaterial 1. Details on statistical analysis

The data are constructed as follows. The data structure of antidepressant prescriptions in the female population aged 40–44 and 45–49 years in Ulsan and Gwangju is shown as example. As shown, we built time-series data with repeated cross-sectional data of psychotropic medication prescriptions. The number of prescriptions by subpopulation (categorized by sex and age) in Ulsan and Gwangju (as a control population) was summarized weekly.

Prescription	Group	Sex	Age	Population	Month	Time	Event
333	Ulsan	Female	40–44	43,627	2	1	0
233	Ulsan	Female	40–44	43,743	3	2	0
…	…	…	…	…	…	…	…
100	Ulsan	Female	40–44	45,105	7	72	1
…	…	…	…	…	…	…	…
433	Ulsan	Female	40–44	46,129	11	141	1
200	Ulsan	Female	40–44	46,129	11	142	1
333	Ulsan	Female	45–49	52,322	2	1	0
267	Ulsan	Female	45–49	52,079	3	2	0
…	…	…	…	…	…	…	…
267	Ulsan	Female	45–49	48,488	7	72	1
…	…	…	…	…	…	…	…
367	Ulsan	Female	45–49	45,710	11	141	1
367	Ulsan	Female	45–49	45,710	11	142	1
400	Gwangju	Female	40–44	58,187	2	1	0
267	Gwangju	Female	40–44	58,280	3	2	0
…	…	…	…	…	…	…	…
200	Gwangju	Female	40–44	58,332	7	72	1
…	…	…	…	…	…	…	…
200	Gwangju	Female	40–44	58,244	11	141	1
467	Gwangju	Female	40–44	58,244	11	142	1
500	Gwangju	Female	45–49	67,346	2	1	0
200	Gwangju	Female	45–49	67,128	3	2	0
…	…	…	…	…	…	…	…
367	Gwangju	Female	45–49	63,363	7	72	1
…	…	…	…	…	…	…	…
267	Gwangju	Female	45–49	60,728	11	142	1
333	Gwangju	Female	45–49	60,728	11	142	1

To confirm the prescription rate difference of the experimental population compared to that of the control, we used a controlled interrupted time series model. The basic form of the regression is as follows.

$$Y_{ijt} = \beta_0 + \beta_1 time_t + \beta_2 event_t + \beta_3 time_t * event_t + \beta_4 group_i + \beta_5 group_i * time_t \\ + \beta_6 group_i * event_t + \beta_7 group_i * time_t * event_t + e_{ijt}$$

i: group; j: characteristic of subpopulation (i.e., sex and age); t: time

Y_{ijt}: Number of weekly prescriptions.
$time_t$: Time (as number of weeks) since the start of follow-up. 1 … 142.
$event_t$: 0 (before earthquake), 1 (after earthquake). The time of the earthquake is 72.
$group_i$: Ulsan or control.
e_{ijt}: Error term.

β_6 indicates the difference of level change between groups, and β_7 indicates the difference of trend change between groups. For interpretation, the time used for estimating β_7 was centered on the onset of the earthquake. The statistical significance of each coefficient was tested.

Additionally, we adjusted for sex and age characteristics in each subpopulation. To account for seasonality, the harmonic function was used. To model rate difference, the logarithm of sex and age-specific population number is used as offset. Because the population structure of Ulsan and controls were different, each cities' sex and age-

specific population was included as the offset term for standardization. The size of the sex and age-specific population is recorded monthly.

The rate difference was fitted using a quasi-Poisson distribution with log-link. Due to the autocorrelation, the confidence interval was estimated by block bootstrapping. The model fitness including the quadratic function of time was compared via a likelihood test; however, the result was non-significant.

After confirming the changes, we estimated the overall changes in weekly prescriptions one year after the earthquake. The interrupted times-series model was constructed based on a non-earthquake scenario using data from Ulsan. The basic model was constructed as follows. Sex age, and seasonality were also adjusted. For interpretation, the time used for estimating β_3 was centered on the onset of the earthquake

$$Y_{ijt} = \beta_0 + \beta_1 time_t + \beta_2 event_t + \beta_3 time_t * event_t$$

j: characteristic of unit (i.e., sex and age); t: time

We estimated the overall changes by calculating β_2 plus β_3 multiplied by the time after the earthquake (i.e., 52 weeks). The rate changes were fitted using a quasi-Poisson distribution with log-link. The absolute changes in volumes (i.e., the number of weekly prescriptions) was compared via Gaussian distribution. The confidence interval was estimated by block bootstrapping. In the subgroup analysis, the significance of the interaction with subpopulation was tested via a likelihood test. After confirming their significance, the overall changes of each subpopulation were estimated.
