# Supplementary material for: The Impact of Moderate Earthquakes on Antidepressant Prescriptions in Ulsan, South Korea: A Controlled Interrupted Time Series Analysis
Source: J Epidemiol. 2023 Dec 5;33(12):600–6. doi: 10.2188/jea.JE20220171 (PMC10635813; doi:10.2188/jea.JE20220171)
Supplement: Supplementary file 1 [file je-33-600-s001.zip › JE20220171/JE20220171_eTables_eFigures_accepted_33-12-clean.pdf]

eTable 1. The list of psychotropic medications included in the study and corresponding anatomical therapeutic chemical code

ATC code	Name
N06A	Antidepressant
N06AA	Tricyclic antidepressant
N06AA02	Imipramine
N06AA04	Clomipramine
N06AA09	Amitriptyline
N06AA10	Nortriptyline
N06AA12	Doxepin
N06AA16	Dosulepin
N06AA17	Amoxapine
N06AA23	Quinupramine
N06AB	Selective serotonin reuptake inhibitors
N06AB03	Fluoxetine
N06AB04	Citalopram
N06AB05	Paroxetine
N06AB06	Sertraline
N06AB08	Fluvoxamine
N06AB10	Escitalopram
N06AG	Monoamine oxidase A inhibitors
N06AG02	Moclobemide
N06AX	Other antidepressants
N06AX05	Trazodone
N06AX11	Mirtazapine
N06AX12	Bupropion
N06AX14	Tianeptine
N06AX16	Venlafaxine
N06AX17	Milnacipran
N06AX21	Duloxetine
N06AX22	Agomelatine
N06AX23	Desvenlafaxine
N06AX25	Hyperici herba
N06AX26	Vortioxetine
N05BA	Benzodiazepine derivatives
N05BA01	Diazepam
N05BA02	Chlordiazepoxide
N05BA05	Potassium clorazepate
N05BA06	Lorazepam
N05BA08	Bromazepam
N05BA09	Clobazam
N05BA12	Alprazolam
N05BA14	Pinazepam
N05BA18	Ethyl loflazepate
N05BA19	Etizolam
N05BA21	Clotiazepam

N05BA23	Tofisopam
N05CD	Benzodiazepine derivatives
N05CD01	Flurazepam
N05CD03	Flunitrazepam
N05CD05	Triazolam
N05CD08	Midazolam
N05CD09	Brotizolam
N05CF02	Zolpidem

ATC, anatomical therapeutic chemical.

eTable 2. Estimated level and trend changes in weekly psychotropic medications prescriptions in ratio of rate ratios compared to the controls

		Antidepressants		Benzodizepines		Zolpidem	
		Level change	Trend change	Level change	Trend change	Level change	Trend change
Main analysis	Ulsan vs Gwangju	1.053 (0.991–1.139)	1.004 (1.002–1.006)	1.013 (0.955–1.064)	1.001 (1.000–1.003)	1.111 (1.019–1.243)	1.001 (0.998–1.003)
Sensitivity analysis 1.	Ulsan vs Daejeon	1.049 (0.988–1.126)	1.004 (1.002–1.006)	1.031 (0.985–1.096)	1.000 (0.999–1.001)	1.033 (0.941–1.170)	1.000 (0.998–1.003)
	Ulsan vs Incheon	1.063 (1.005–1.138)	1.003 (1.002–1.005)	1.035 (0.979–1.104)	0.999 (0.998–1.001)	1.044 (0.969–1.141)	0.999 (0.997–1.001)
Sensitivity analysis 2.	Ulsan vs Gwangju	1.086 (0.978–1.159)	1.003 (1.001–1.005)	1.012 (0.934–1.061)	1.001 (0.998–1.001)	1.031 (0.900–1.084)	1.000 (0.997–1.002)
Sensitivity analysis 3.	Ulsan vs Gwangju	1.054 (1.002–1.115)	1.003 (1.002–1.004)	—	—	—	—
	Ulsan vs Daejeon	1.056 (0.979–1.145)	1.004 (1.002–1.005)	—	—	—	—
	Ulsan vs Incheon	1.077 (1.016–1.157)	1.003 (1.002–1.005)	—	—	—	—

The changes in level and trends are estimated with ratio of rate ratios. The estimates represent weekly changes. Gwangju was set as control group in the main analysis. In sensitivity analysis 1, other metropolitan cities were used as control groups. In sensitivity analysis 2, it was assumed that residents were affected since second earthquake on September 12, 2016. In sensitivity analysis 3, antidepressant prescriptions for cerebrovascular disease, epilepsy, dementia, or Parkinson's disease were excluded. Reference group was set as control group. The changes are presented with 95% confidence intervals.

eTable 3. Estimated level and trend changes in weekly psychotropic medications prescriptions in controls

	Antidepressants		Benzodizepines		Zolpidem	
	Level change	Trend change	Level change	Trend change	Level change	Trend change
Gwangju	0.978 (0.924–1.037)	1.001 (0.999–1.002)	0.969 (0.935–1.038)	0.999 (0.998–1.001)	0.844 (0.767–0.933)	0.998 (0.996–1.001)
Daejeon	0.981 (0.935–1.049)	1.001 (0.9996–1.003)	0.953 (0.898–1.002)	1.001 (0.999–1.003)	0.907 (0.851–0.974)	0.999 (0.997–1.001)
Incheon	0.968 (0.909–1.029)	1.001 (0.9998–1.003)	0.950 (0.890–1.003)	1.001 (0.9999–1.003)	0.898 (0.835–0.959)	1.000 (0.998–1.002)

The changes in level and trends are estimated with rates ratio. The estimates represent weekly changes. The changes are presented with 95% confidence intervals. None of the results were statistically significant ($p > 0.05$), except the level change in zolpidem prescriptions.

eTable 4. Estimated level and trend changes in weekly antidepressant prescriptions in rate ratio and absolute volumes in Ulsan compared to non-earthquake scenario

		Rate ratio			Absolute volumes		
		Trend	Level change	Trend change	Trend	Level change	Trend change
**Ulsan**	Total	0.999 (0.997–1.000)	1.030 (0.969–1.136)	1.005 (1.003–1.007)	-4.11 (-14.02 to 4.00)	251.15 (-282.30 to 806.40)	33.43 (21.41–49.61)
**Subpopulations**							
Sex	Female	1.000 (0.999–1.001)	1.020 (0.964–1.104)	1.003 (1.001–1.004)	2.09 (-3.16 to 6.29)	99.61 (-192.53 to 395.26)	10.17 (3.42–18.64)
	Male	0.997 (0.994–0.999)	1.049 (0.952–1.212)	1.008 (1.006–1.012)	-6.20 (-13.47 to -1.26)	151.54 (-170.05 to 532.44)	23.27 (15.76–33.42)
Ages, years	<20	1.001 (0.996–1.009)	1.258 (0.958–1.958)	0.999 (0.989–1.006)	0.15 (-1.02 to 1.77)	70.78 (-14.51 to 163.20)	-0.41 (-2.64 to 1.45)
	20–39	0.994 (0.991–0.997)	1.178 (0.998–1.382)	1.009 (1.006–1.014)	-6.21 (-8.93 to -3.70)	148.04 (-19.52 to 356.03)	9.92 (6.09–14.34)
	40–59	1.000 (0.999–1.002)	1.081 (1.007–1.207)	1.004 (1.003–1.007)	1.57 (-2.64 to 4.52)	222.71 (-50.58 to 508.02)	13.30 (7.66–20.82)
	≥60	0.999 (0.997–1.000)	0.917 (0.855–1.011)	1.004 (1.002–1.007)	0.39 (-5.28 to 4.02)	-190.38 (-462.24 to 53.54)	10.62 (5.53–18.44)
Sex and ages, years	Female and <20	1.001 (0.996–1.010)	0.682 (0.308–1.159)	1.014 (1.002–1.033)	0.00 (-0.45 to 0.91)	-37.12 (-111.55 to 9.67)	1.39 (0.09–2.93)
	Female and 20–39	0.995 (0.990–0.999)	1.113 (0.890–1.354)	1.010 (1.005–1.014)	-3.23 (-5.74 to -0.88)	39.40 (-87.35 to 167.67)	5.64 (3.04–8.62)
	Female and 40–59	1.002 (1.000–1.003)	1.059 (0.953–1.199)	1.000 (0.997–1.003)	2.91 (0.20–5.48)	117.61 (-94.96 to 322.22)	-0.46 (-5.02 to 5.28)
	Female and ≥60	1.000 (0.998–1.001)	0.973 (0.901–1.082)	1.002 (1.000–1.004)	2.41 (-0.83 to 5.13)	-20.28 (-182.49 to 132.22)	3.59 (-0.35 to 8.75)
	Male and <20	1.002 (0.990–1.012)	1.823 (1.373–4.450)	0.989 (0.974–1.002)	0.15 (-1.06 to 1.48)	107.90 (50.88–181.81)	-1.80 (-3.56 to -0.30)
	Male and 20–39	0.994 (0.991–0.997)	1.260 (1.043–1.529)	1.009 (1.004–1.015)	-2.98 (-4.57 to -1.27)	108.64 (4.73–203.94)	4.28 (1.88–6.95)
	Male and 40–59	0.998 (0.995–1.000)	1.138 (0.992–1.363)	1.011 (1.009–1.016)	-1.34 (-4.52 to 1.00)	105.10 (-75.56 to 282.44)	13.76 (10.61–18.65)
	Male and ≥60	0.997 (0.995–0.998)	0.810 (0.735–0.927)	1.007 (1.004–1.011)	-2.02 (-4.00 to -0.05)	-170.10 (-286.81 to -61.23)	7.03 (4.35–10.61)

The changes in level and trends are estimated with rates ratio and difference in an absolute volume. The estimates represent weekly changes. The amount of absolute volumes are scaled to present whole population changes. The changes are presented with 95% confidence intervals.

eFigure 1. Population characteristics of Ulsan and controls at the start (February 2015) and the end (November 2017) of the study period

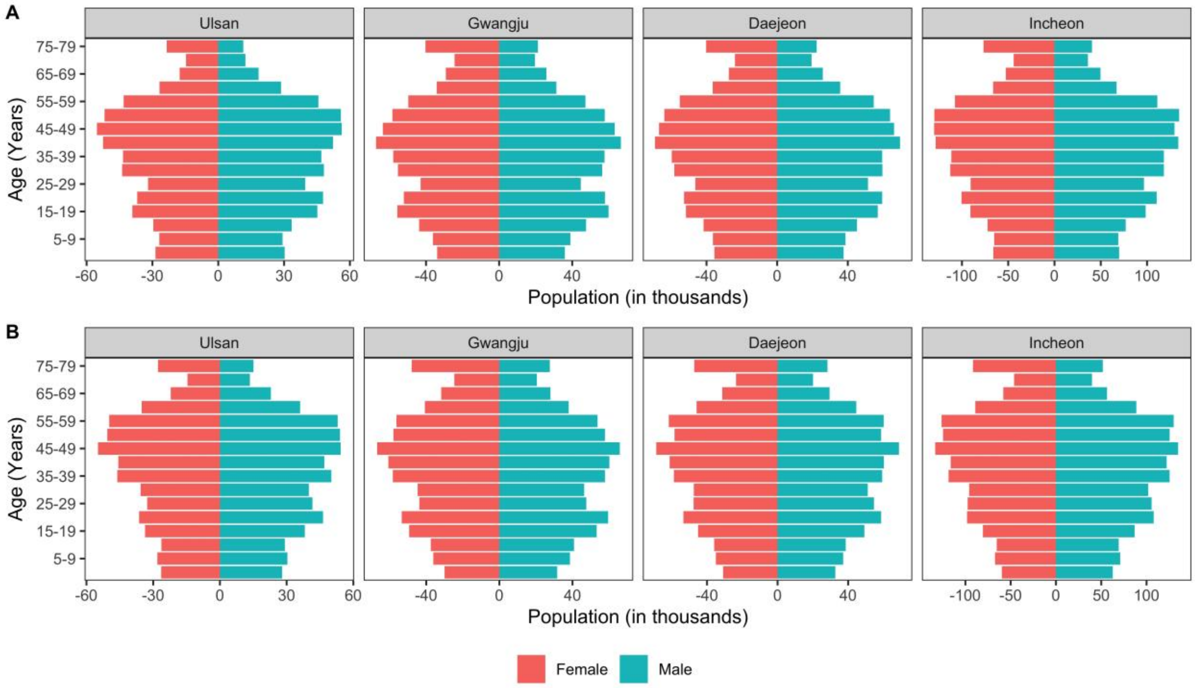

The population characteristics of Ulsan and controls are presented in population pyramids. Panel A shows the characteristics at the start of the study (February 2015). Panel B displays the characteristics at the end of the study (November 2017).

eFigure 2. Observed and estimated volume of weekly prescribed psychotropic medications in Ulsan and control (Daejeon) during the study periods

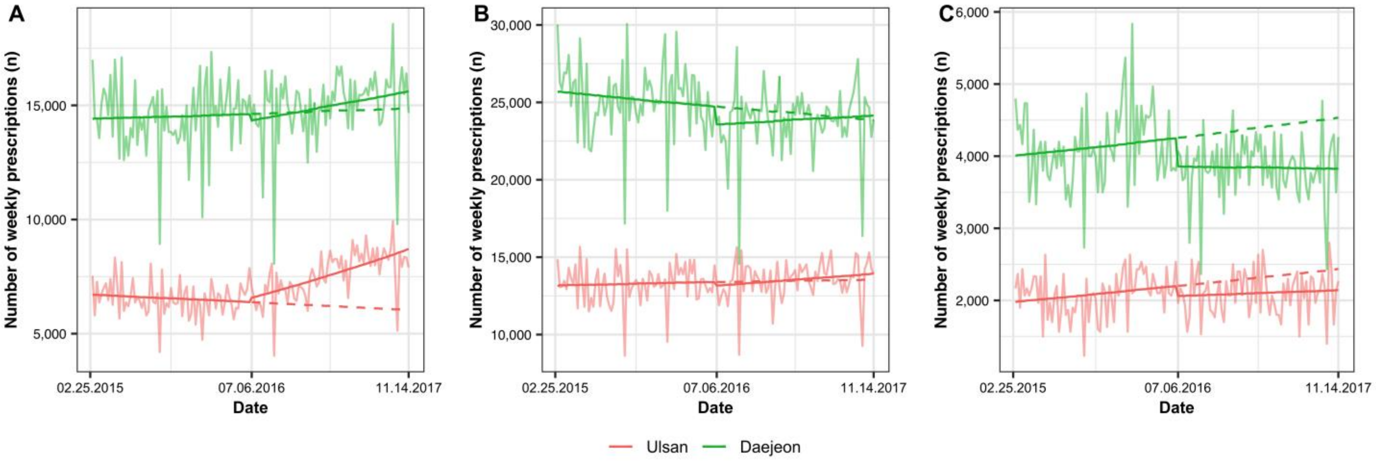

Daejeon was selected as a control group for sensitivity analysis. The series of earthquakes starts in the July 5, 2016. The solid line is modelled data and the dashed line is counterfactual data with assumption that the earthquakes did not occur. The modelled data are de-seasonalized. Translucent lines are observed data. The trend changes in control group were all non-significant. A: Antidepressants; B: Benzodiazepines; C: Zolpidem

eFigure 3. Observed and estimated volume of weekly prescribed psychotropic medications in Ulsan and control (Incheon) during the study periods

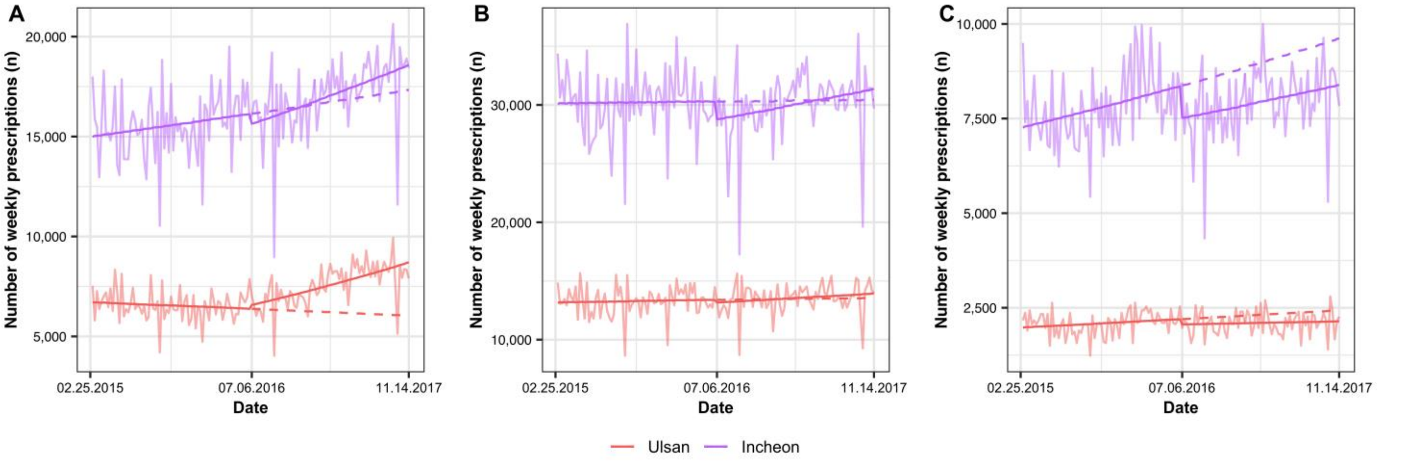

Incheon was selected as a control group for sensitivity analysis. The first earthquake occurred on July 5, 2016. The solid line is modelled data and the dashed line is counterfactual data with assumption that the earthquakes did not occur. The modelled data are de-seasonalized. Translucent lines are observed data. The trend changes in control group were all non-significant. A: antidepressants; B: benzodiazepines; C: zolpidem.

eFigure 4. Observed and estimated volume of weekly prescribed psychotropic medications in Ulsan and control during the study periods with a different way of defining the event

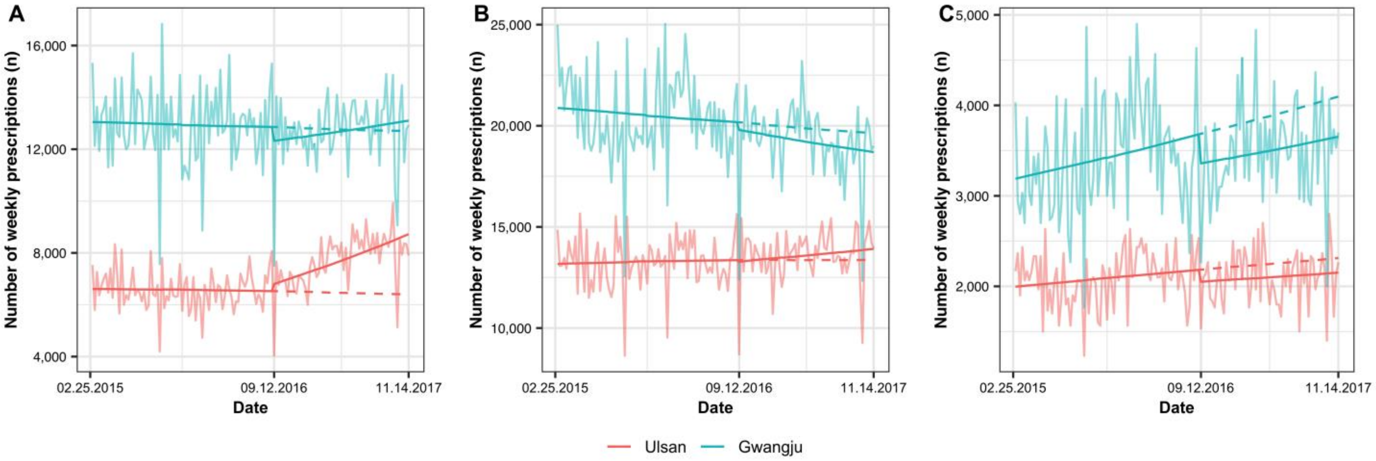

It was assumed that Ulsan residents were affected since the second earthquake on September 12, 2016. The solid line is modelled data and the dashed line is counterfactual data with assumption that the earthquakes did not occur. The modelled data are de-seasonalized. Translucent lines are observed data. A: antidepressants; B: benzodiazepines; C: zolpidem.

eFigure 5. Observed and estimated volume of weekly prescribed antidepressant in Ulsan and controls during the study periods excluding patients with cerebrovascular disease, epilepsy, dementia, or Parkinson's disease

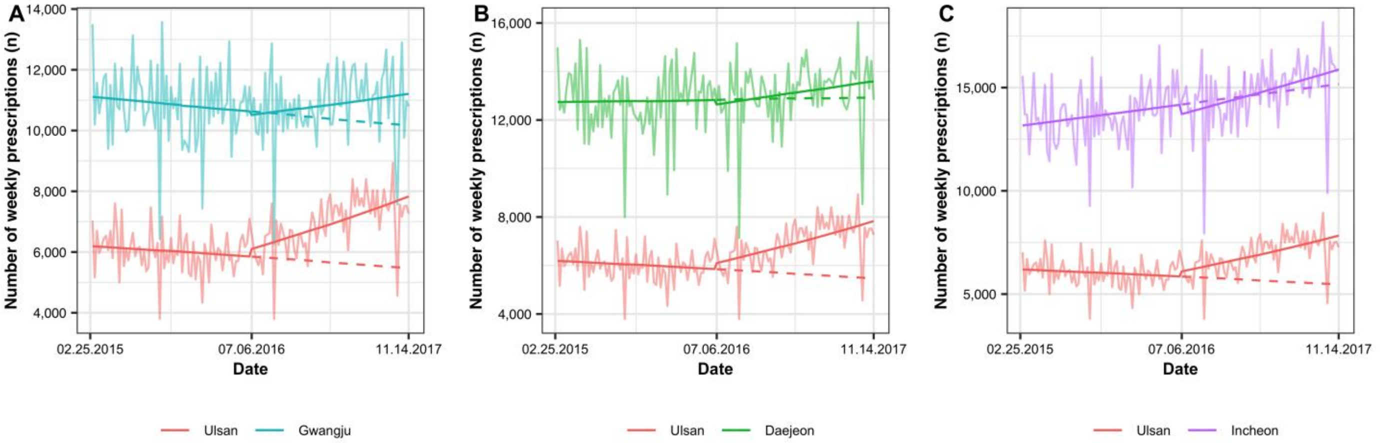

Antidepressant prescriptions for patients with cerebrovascular disease, epilepsy, dementia, or Parkinson's disease were excluded from analysis. The solid line is modelled data and the dashed line is counterfactual data with assumption that the earthquakes did not occur. The modelled data are de-seasonalized. Translucent lines are observed data. A: Gwangju as control; B: Daejeon as control; C: Incheon as control.
